# Quantitative EEG as a biomarker in mild cognitive impairment with Lewy bodies

**DOI:** 10.1186/s13195-020-00650-1

**Published:** 2020-07-08

**Authors:** Julia Schumacher, John-Paul Taylor, Calum A. Hamilton, Michael Firbank, Ruth A. Cromarty, Paul C. Donaghy, Gemma Roberts, Louise Allan, Jim Lloyd, Rory Durcan, Nicola Barnett, John T. O’Brien, Alan J. Thomas

**Affiliations:** 1 Translational and Clinical Research Institute, Faculty of Medical Sciences, Newcastle University, Biomedical Research Building 3rd floor, Campus for Ageing and Vitality, Newcastle upon Tyne, NE4 5PL UK; 2 Nuclear Medicine Department, Newcastle upon Tyne Hospitals NHS Foundation Trust, Newcastle upon Tyne, UK; 3 Institute of Health Research, University of Exeter, Exeter, UK; 4 Department of Psychiatry, University of Cambridge School of Medicine, Cambridge, CB2 0SP UK

**Keywords:** Dementia with Lewy bodies, Alzheimer’s disease, Quantitative electroencephalography, Biomarker

## Abstract

**Objectives:**

To investigate using quantitative EEG the (1) differences between patients with mild cognitive impairment with Lewy bodies (MCI-LB) and MCI with Alzheimer’s disease (MCI-AD) and (2) its utility as a potential biomarker for early differential diagnosis.

**Methods:**

We analyzed eyes-closed, resting-state, high-density EEG data from highly phenotyped participants (39 MCI-LB, 36 MCI-AD, and 31 healthy controls). EEG measures included spectral power in different frequency bands (delta, theta, pre-alpha, alpha, and beta), theta/alpha ratio, dominant frequency, and dominant frequency variability. Receiver operating characteristic (ROC) analyses were performed to assess diagnostic accuracy.

**Results:**

There was a shift in power from beta and alpha frequency bands towards slower frequencies in the pre-alpha and theta range in MCI-LB compared to healthy controls. Additionally, the dominant frequency was slower in MCI-LB compared to controls. We found significantly increased pre-alpha power, decreased beta power, and slower dominant frequency in MCI-LB compared to MCI-AD. EEG abnormalities were more apparent in MCI-LB cases with more diagnostic features. There were no significant differences between MCI-AD and controls. In the ROC analysis to distinguish MCI-LB from MCI-AD, beta power and dominant frequency showed the highest area under the curve values of 0.71 and 0.70, respectively. While specificity was high for some measures (up to 0.97 for alpha power and 0.94 for theta/alpha ratio), sensitivity was generally much lower.

**Conclusions:**

Early EEG slowing is a specific feature of MCI-LB compared to MCI-AD. However, there is an overlap between the two MCI groups which makes it difficult to distinguish between them based on EEG alone.

## Background

The early diagnosis of dementia is becoming increasingly important as it is likely that potential disease-modifying treatments will have their greatest effect at this stage. Additionally, accurate and early differential diagnosis of dementia subtypes is crucial for correct patient stratification for clinical trials and research studies and for optimizing clinical care such as by highlighting the need to carefully avoid using medication with anticholinergic properties and encouraging active identification of therapeutic targets such as orthostatic hypotension and constipation [1].

The intermediate stage between normal aging and dementia in which cognitive decline is present, but independence in activities of daily living is still preserved, is referred to as mild cognitive impairment (MCI) [2, 3]. While much research has focused on MCI patients who later develop Alzheimer’s disease (AD), MCI in the context of dementia with Lewy bodies (DLB) has only been characterized more recently with research criteria recently published [4–6]. Differential diagnosis is often impeded by a substantial clinical overlap between AD and DLB, which is especially pronounced in early stages, highlighting the need for objective biomarkers. In DLB, dopaminergic brain imaging and myocardial scintigraphy are indicative biomarkers [7]; these can improve diagnostic accuracy at the MCI stage [8] and are included in the new research criteria for MCI with Lewy bodies (MCI-LB). EEG has been suggested as a non-invasive alternative, and an abnormal EEG is listed as a supportive biomarker in the DLB diagnostic criteria [7]. However, its value for differential diagnosis at the MCI stage is less certain.

The aims of the present study were therefore to investigate changes in quantitative EEG measures in a large cohort of well-characterized patients, all of whom had biomarker assessments with both dopaminergic and cardiac MIGB imaging, and to test the utility of EEG as a biomarker for early differential diagnosis.

The secondary aim was to better understand the relationship between EEG abnormalities in the MCI-LB group and the core Lewy body symptoms. We hypothesized that EEG abnormalities would be more severe in patients with more core symptoms.

## Methods

### Participants

All participants in this study were over 60 years of age. Patients with a clinical diagnosis of MCI in memory services in the north east of England were screened and approached for this study if they reported additional clinical symptoms suggestive of Lewy body disease (e.g., mood changes, sleep disturbances, or autonomic symptoms) or any core DLB features (visual hallucinations, cognitive fluctuations, parkinsonism, and REM sleep behavior disorder). After obtaining written informed consent, participants underwent a baseline clinical assessment including the Addenbrooke’s Cognitive Examination–Revised (ACE-R), from which the Mini-Mental State Examination (MMSE) score was derived; the Unified Parkinson’s Disease Rating Scale motor sub-score (UPDRS-III); the Epworth Sleepiness Scale (ESS); and the Geriatric Depression Scale (GDS). Additionally, the Instrumental Activities of Daily Living (IADL) Scale, the Clinician Assessment of Fluctuations (CAF), the Dementia Cognitive Fluctuations Scale (DCFS), the North-East Visual Hallucinations Interview (NEVHI), the Neuropsychiatric Inventory (NPI), and the Mayo Sleep Questionnaire (MSQ) were administered to informants, and the Clinical Dementia Rating (CDR) Scale and the Cumulative Illness Rating Scale for Geriatrics (CIRS-G) were completed based on the clinical history and research assessments. In addition to a detailed clinical assessment, participants had already undergone dopaminergic imaging with ^123^I-*N*-fluoropropyl-2β-carbomethoxy-3β-(4-iodophenyl) single-photon emission computed tomography (FP-CIT SPECT) and ^123^iodine-metaiodobenzylguanidine (MIBG) myocardial scintigraphy through their involvement in an ongoing study investigating the diagnostic accuracy of imaging biomarkers in MCI, and this information was used to apply diagnostic criteria (see below).

After the initial baseline visit, participants were followed annually for up to 3 years with a mean follow-up time of 16.5 months (standard deviation = 6.5 months). Patients who were taking dopaminergic medication were assessed in the “ON” motor state.

### Diagnosis

Diagnoses were based on all information available at the end of the recruitment period (December 2019) including any baseline and follow-up visits. MCI diagnoses were made independently by a consensus panel of three experienced old-age psychiatrists (AJT, PCD, JPT) in accordance with NIA-AA criteria [3], i.e., subjective and objective cognitive impairment with maintained independence of function with minimal aids or assistance and a CDR of 0 or 0.5. Patients with a diagnosis of dementia were excluded from the study. Furthermore, patients with possible contributing frontotemporal or vascular etiologies or with a history of parkinsonism of more than 1 year prior to the onset of cognitive impairment were excluded. The presence or absence of the core Lewy body symptoms was rated by the panel utilizing the rating scales and all information from the clinical assessments [7]. Findings from the FP-CIT and MIBG scans were used for diagnosis (see below), but the clinical MCI diagnoses as well as the rating of the presence/absence of core DLB symptoms were performed blind to these imaging findings.

A diagnosis of MCI with probable Alzheimer’s disease (MCI-AD) was given to patients who had no core Lewy body symptoms, negative FP-CIT and MIBG findings, and evidence of cognitive decline that was characteristic of AD, i.e., they met the additional NIA-AA criterion for “etiology of MCI consistent with AD pathophysiologic process” [3].

Probable MCI with Lewy bodies (MCI-LB) was diagnosed if a patient had two or more core Lewy body symptoms or one core symptom in addition to a positive FP-CIT or MIBG scan [6].

**Fig. 1:**
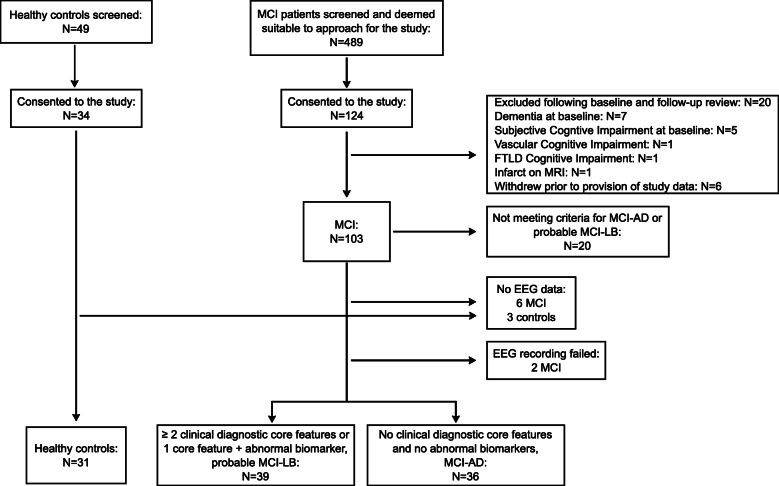
Study flowchart

Out of 103 MCI participants who were included in the study, 20 had only one core Lewy body symptom with negative FP-CIT and MIBG scans or no core symptoms in addition to a positive FP-CIT or MIBG scan, i.e., they did not meet the criteria for either MCI-AD or probable MCI-LB and were therefore not included in the present analysis (Fig. 1). Additionally, eight MCI participants did not have usable EEG data available. This study therefore included 39 participants who were diagnosed with probable MCI-LB and 36 who were diagnosed with MCI-AD. Healthy control participants (*N* = 31) were recruited from relatives and friends of patients and from a local research register. Control participants had the same assessment as the patients and had no history of psychiatric or neurological illness and no evidence of any cognitive decline. They also had normal structural MR imaging. The study was approved by the local ethics committee, and written informed consent was obtained from all participants.

### EEG acquisition and pre-processing

Resting-state high-density EEG recordings were acquired from all participants using Waveguard caps (ANT Neuro, The Netherlands) comprising 128 sintered Ag/AgCl electrodes that were placed according to the 10-5 system. Participants were seated during the recording and instructed to remain awake. Electrode impedance was kept below 5 kΩ, and continuous EEG data were recorded at a sampling frequency of 1024 Hz. Three hundred seconds of eyes-closed data were recorded from each participant. Participants were supervised by the EEG technician during the recording to monitor adherence to the protocol. The ground electrode was attached to the right clavicle, and all EEG channels were referenced to Fz during recording.

Pre-processing of eyes-closed EEG data was performed using the EEGLAB toolbox (version 14) in MATLAB (R2017a) [9] and was blinded to group membership. First, EEG data were bandpass-filtered between 0.3 and 54 Hz using a second-order Butterworth filter and split into non-overlapping epochs of 2 s. Subsequently, EEG recordings were visually inspected to identify noisy channels and noisy epochs which were excluded prior to applying independent component analysis for further artifact removal. The resulting components were visually inspected, and components representing muscular, cardiac, ocular, or electrical (50 Hz line noise) artifacts were rejected. The previously excluded channels were then replaced using spherical spline interpolation, and data were recomputed against the average reference. For each participant, the first 45 2-s-long artifact-free epochs were selected for further analysis.

### Frequency analysis

For each 2-s epoch, the power spectral density (PSD) was estimated using Bartlett’s method in MATLAB (R2017a) with a frequency resolution of 0.5 Hz and a Hamming window across the power spectrum from 2 to 30 Hz, for each electrode separately. To compensate for inter-individual variability in brain neurophysiology, anatomy, and physical tissue properties, the PSD was normalized by the total power across the power spectrum [10].

For each electrode separately, the mean power across all included epochs was estimated for different standard EEG frequency bands including delta (2–4 Hz), theta (4–5.5 Hz), pre-alpha (5.5–8 Hz), alpha (8–13 Hz), and beta (13–30 Hz). Higher frequencies were excluded because these are particularly affected by muscular artifacts [11]. The dominant frequency was calculated as the frequency with the highest power between 4 and 15 Hz (averaged across epochs). Dominant frequency was calculated for all electrodes as well as from occipital electrodes only (PO9, PO7, POO9h, PO5, O1, PO3, POO3h, OI1h, POz, Oz, PO4, POO4h, PO6, O2, OI2h, PO8, POO10h, PO10). Dominant frequency variability was defined as the standard deviation of dominant frequency across epochs [12].

### Statistics

Statistical analyses were performed in SPSS and R (https://www.r-project.org/). Relative power within the different frequency bands was compared between the three groups using a multivariate ANOVA with a within-subject factor of frequency band and a between-subject factor of diagnosis, followed by univariate ANOVAs and post hoc tests, Bonferroni-corrected for multiple comparisons. Since relative power was not normally distributed in all groups, the variables were log-transformed before applying the ANOVA. Theta/alpha ratio, dominant frequency, and dominant frequency variability were compared between the groups using univariate ANOVAs followed by post hoc tests (Bonferroni-corrected). To account for differences in the number of male and female participants in the three groups, sex was included as a covariate in all analyses.

To test the applicability of this analysis to a clinical setting, the group comparison of relative power within the different frequency bands was repeated only including the 21 electrodes that are part of the 10-20 system that is routinely used in clinical practice.

Additionally, to test whether EEG changes have a different spatial distribution in the different groups, we conducted a supplementary analysis splitting the whole set of electrodes into four macroscopic regions (frontal, central, lateral, and posterior) and applying a repeated measures ANOVA with region as the within-subject factor and diagnosis as the between-subject factor [10]. If the interaction between region and diagnosis was significant, this was followed up by post hoc univariate ANOVAs.

A receiver operating characteristic (ROC) analysis was conducted in R to assess the sensitivity and specificity of the different EEG measures to distinguish between MCI-AD and MCI-LB patients. The sensitivity/specificity cutoff was determined using Youden’s index.

To assess the association between the range of Lewy body symptomatology and EEG abnormalities, we performed an exploratory analysis in which we compared quantitative EEG measures between MCI-LB patients who had two core symptoms or one core symptom and one abnormal biomarker (*N* = 13) with those patients who had more than two core symptoms/abnormal biomarkers (*N* = 26) using two-sample *t* tests. Furthermore, Spearman’s correlations between symptom/biomarker count (ranging from 2 to 6) and the different EEG measures were computed in the MCI-LB group.

The association between quantitative EEG measures and overall cognitive impairment was assessed using Spearman’s correlations, in the MCI-AD and MCI-LB groups separately. *p* values were FDR-corrected for multiple comparisons.

Additionally, we investigated the association between EEG characteristics and the core Lewy body symptoms of visual hallucinations and cognitive fluctuations which have been shown to be related to EEG abnormalities in dementia patients [10, 13, 14]. To this end, two-sample *t* tests were performed, dichotomizing the MCI-LB group according to the presence/absence of visual hallucinations and cognitive fluctuations.

Given previous reports of an effect of acetylcholinesterase inhibitors on the EEG signal [15, 16], a two-sample *t* test was performed in the MCI-LB group to compare the EEG characteristics between patients who were taking acetylcholinesterase inhibitors and those patients not taking these medications.

## Results

### Demographics

All groups were similar in age (Table 1). The proportion of male participants was higher in the MCI-LB group whereas more MCI-AD patients were female. MCI-LB patients had significantly lower years of education compared to controls, but there was no significant difference between the two MCI groups in terms of education. More MCI-LB patients were taking cholinesterase inhibitors and Parkinson’s disease medication compared to MCI-AD patients. The two MCI groups were matched in terms of overall cognitive impairment. As expected, the MCI-LB group had more parkinsonism, higher cognitive fluctuation, and visual hallucination scores compared to the MCI-AD group.

**Table 1:** Demographic and clinical variables, mean (standard deviation)

	HC (*N* = 31)	MCI-AD (*N* = 36)	MCI-LB (*N* = 39)	Group differences
Male to female	22:9	15:21	35:4	*χ*^2^ = 20.0, *p* < 0.001^a^ *p*(HC,MCI-AD) = 0.02 *p*(HC,MCI-LB) = 0.045 *p*(MCI-AD,MCI-LB) < 0.001
Age	73.7 (7.3)	76.1 (7.7)	74.7 (6.4)	*F*(2, 103) = 1.0, *p* = 0.38^b^
AChEI	–	7 (19%)^e^	18 (46%)^f^	*χ*^2^ = 5.7, *p* = 0.02^c^
PD meds	–	0^e^	4 (10%)^f^	*χ*^2^ = 3.8, *p* = 0.052^c^
Years of education	14.7 (4.0)^g^	12.9 (3.4)^h^	12.1 (2.8)	*F*(2, 100) = 5.1, *p* = 0.008^c^ *p*(HC,MCI-AD) = 0.12 *p*(HC,MCI-LB) = 0.006 *p*(MCI-AD,MCI-LB) = 0.87
ACE-R	92.7 (4.2)	82.4 (8.5)	83.8 (9.2)	*t*_73_ = 0.7, *p* = 0.50^d^
MMSE	28.5 (1.1)	26.9 (2.1)	26.6 (2.5)	*t*_73_ = 0.7, *p* = 0.51^d^
UPDRS III	5.5 (4.4)	15.1 (13.8)	23.3 (14.2)	*t*_73_ = 2.5, *p* = 0.01^d^
DCFS	–	6.9 (1.9)^i^	8.5 (3.3)^k^	*t*_61_ = 2.4, *p* = 0.02^d^
CAF total	–	1.4 (2.7)^i^	3.7 (4.2)^k^	*t*_61_ = 2.5, *p* = 0.02^d^
NPI total	–	8.6 (9.3)^i^	16.4 (12.9)^k^	*t*_61_ = 2.7, *p* = 0.01^d^
NEVHI	–	0.8 (1.5)^e^	2.7 (4.1)	*t*_71_ = 2.6, *p* = 0.01^d^
GDS	1.3 (1.8)	3.5 (2.5)	5.1 (4.1)	*F*(2, 103) = 13.4, *p* < 0.001^b^ *p*(HC,MCI-AD) = 0.01 *p*(HC,MCI-LB) < 0.001 *p*(MCI-AD,MCI-LB) = 0.08

*ACE-R* Addenbrooke’s Cognitive Examination–Revised, *AChEI* number of patients taking acetylcholinesterase inhibitors, *CAF total* clinician assessment of fluctuation total score, *DCFS* Dementia Cognitive Fluctuation Scale, *GDS* Geriatric Depression Scale, *HC* healthy controls, *MCI-AD* mild cognitive impairment with Alzheimer’s disease, *MCI-LB* probable mild cognitive impairment with Lewy bodies, *MMSE* Mini-Mental State Examination, *NEVHI* North-East Visual Hallucinations Interview, *NPI* Neuropsychiatric Inventory, *PD meds* number of patients taking dopaminergic medication for the management of Parkinson’s disease symptoms, *UPDRS III* Unified Parkinson’s Disease Rating Scale III (motor subsection)

^a^Chi-square test HC, MCI-AD, MCI-LB

^b^Univariate ANOVA HC, MCI-AD, MCI-LB

^c^Chi-square test MCI-AD, MCI-LB

^d^Student’s *t* test MCI-AD, MCI-LB

^e^*N* = 34

^f^*N* = 38

^g^*N* = 29

^h^*N* = 35

^i^*N* = 27

^k^*N* = 36

### EEG frequency analysis

**Fig. 2:**
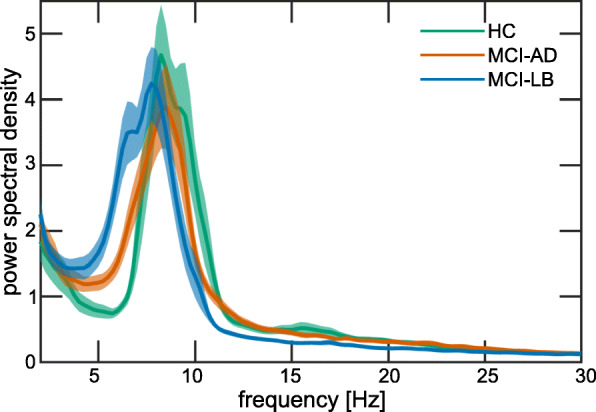
Mean power spectra for the three diagnostic groups. Shaded areas indicate standard errors. HC, healthy controls; MCI-AD, mild cognitive impairment with Alzheimer’s disease; MCI-LB, probable mild cognitive impairment with Lewy bodies

**Fig. 3:**
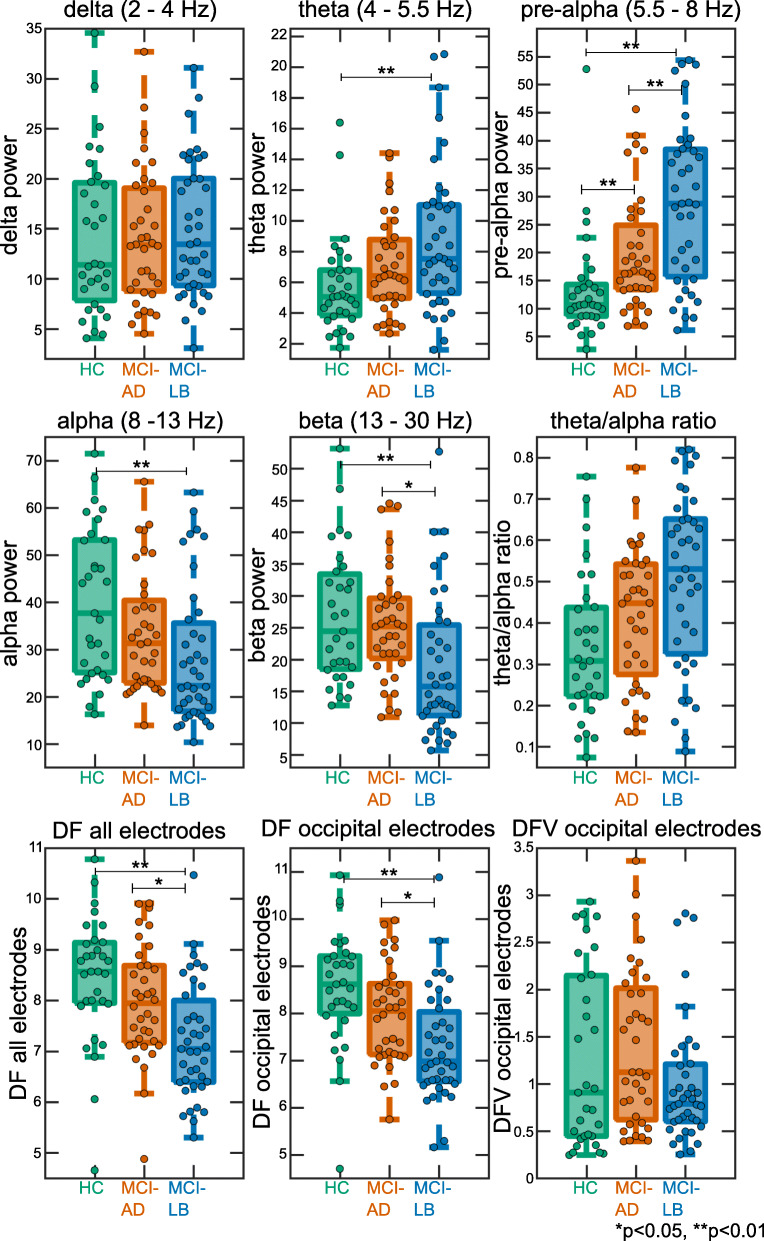
Group comparison of quantitative EEG characteristics. In each boxplot, the central line corresponds to the sample median; the upper and lower border of the box represent the 25th and 75th percentile, respectively; and the length of the whiskers is 1.5 times the interquartile range. Corresponding results from statistical comparisons between the groups are presented in Table 2. DF, dominant frequency; DFV, dominant frequency variability; HC, healthy controls; MCI-AD, mild cognitive impairment with Alzheimer’s disease; MCI-LB, probable mild cognitive impairment with Lewy bodies

The multivariate ANOVA revealed an overall effect of diagnosis: *F*(10, 196) = 5.1, *p* < 0.001. Follow-up univariate ANOVAs showed the following (see Table 2 and Figs. 2 and 3): There were no group differences in terms of delta power. Theta power was increased in MCI-LB (mean = 8.8, standard deviation (SD) = 4.8) compared to controls (mean = 5.7, SD = 3.2) with no significant difference between controls and MCI-AD (mean = 7.0, SD = 3.1) or between the two MCI groups. Pre-alpha power was increased in MCI-LB (mean = 28.8, SD = 14.2) and MCI-AD (mean = 19.6, SD = 10.2) compared to controls (mean = 13.3, SD = 9.2), and it was further increased in MCI-LB compared to MCI-AD. Alpha power was decreased in MCI-LB (mean = 28.3, SD = 14.7) compared to controls (mean = 39.9, SD = 15.8) with no difference between controls and MCI-AD (mean = 35.6, SD = 12.7) or between the two MCI groups. However, when the alpha band was split further into low-alpha (8–10 Hz) and high-alpha (10–13 Hz), it became evident that high-alpha power was significantly reduced in MCI-LB compared to both controls (*p* < 0.001) and MCI-AD (*p* < 0.001) with no difference between MCI-AD and controls (*p* = 1.0) while there were no group differences in terms of low-alpha power (*F*(2, 102) = 1.7, *p* = 0.2). Beta power was reduced in MCI-LB (mean = 18.9, SD = 11.0) compared to both controls (mean = 26.7, SD = 10.3) and MCI-AD (mean = 25.4, SD = 8.7), but not different between MCI-AD and controls.

**Table 2:** Group comparison of quantitative EEG characteristics

	HC	MCI-AD	MCI-LB	Group comparison
Delta power	14.1 [11.3, 17.0]	14.1 [11.8, 16.3]	14.9 [12.7, 17.1]	*F*(2, 102) = 0.8, *p* = 0.47
Theta power	5.7 [4.6, 6.9]	7.0 [6.0, 8.1]	8.8 [7.2, 10.3]	*F*(2, 102) = 4.8, *p* = 0.01
				*p*(HC,MCI-AD) = 0.19 *p*(HC,MCI-LB) = 0.004 *p*(MCI-AD,MCI-LB) = 0.46
Pre-alpha power	13.3 [9.9, 16.7]	19.6 [16.1, 23.1]	28.8 [24.2, 33.4]	*F*(2, 102) = 16.0, *p* < 0.001
				*p*(HC,MCI-AD) = 0.006 *p*(HC,MCI-LB) < 0.001 *p*(MCI-AD,MCI-LB) = 0.02
Alpha power	39.9 [34.1, 45.6]	33.6 [29.3, 37.9]	28.3 [23.6, 33.1]	*F*(2, 102) = 5.5, *p* = 0.005
				*p*(HC,MCI-AD) = 0.39 *p*(HC,MCI-LB) = 0.001 *p*(MCI-AD,MCI-LB) = 0.07
Beta power	26.7 [22.9, 30.4]	25.4 [22.4, 28.4]	18.9 [15.3, 22.5]	*F*(2, 102) = 8.1, *p* = 0.001
				*p*(HC,MCI-AD) = 1.0 *p*(HC,MCI-LB) < 0.001 *p*(MCI-AD,MCI-LB) = 0.001
Theta/alpha ratio	0.34 [0.27, 0.40]	0.42 [0.36, 0.47]	0.51 [0.44, 0.57]	*F*(2, 102) = 7.5, *p* < 0.001
				*p*(HC,MCI-AD) = 0.22 *p*(HC,MCI-LB) = 0.001 *p*(MCI-AD,MCI-LB) = 0.10
DF, all electrodes	8.4 [8.0, 8.9]	8.0 [7.6, 8.3]	7.2 [6.8, 7.6]	*F*(2, 102) = 8.7, *p* < 0.001
				*p*(HC,MCI-AD) = 0.29 *p*(HC,MCI-LB) < 0.001 *p*(MCI-AD,MCI-LB) = 0.01
DF, occipital electrodes	8.5 [8.1, 9.0]	8.0 [7.6, 8.3]	7.3 [6.9, 7.7]	*F*(2, 102) = 8.7, *p* < 0.001
				*p*(HC,MCI-AD) = 0.16 *p*(HC,MCI-LB) < 0.001 *p*(MCI-AD,MCI-LB) = 0.03
DFV, occipital electrodes	1.3 [0.9, 1.6]	1.4 [1.1, 1.7]	1.0 [0.8, 1.2]	*F*(2, 102) = 3.6, *p* = 0.03
				*p*(HC,MCI-AD) = 0.54 *p*(HC,MCI-LB) = 1.0 *p*(MCI-AD,MCI-LB) = 0.17

Mean [95% confidence interval] of different quantitative EEG characteristics. Group comparisons were performed using univariate ANOVAs followed by post hoc tests, Bonferroni-corrected for multiple comparisons. Sex was included as a covariate

*DF* dominant frequency, *DFV* dominant frequency variability, *HC* healthy controls, *MCI-AD* mild cognitive impairment with Alzheimer’s disease, *MCI-LB* probable mild cognitive impairment with Lewy bodies

The theta/alpha ratio was increased in MCI-LB (mean = 0.51, SD = 0.21) compared to controls (mean = 0.34, SD = 0.17), but not significantly different between MCI-AD (mean = 0.42, SD = 0.16) and controls or between the two MCI groups.

Dominant frequency was slower in MCI-LB (mean = 7.2, SD = 1.1) compared to both MCI-AD (mean = 8.0, SD = 1.1) and controls (mean = 8.4, SD = 1.2), but there was no significant difference between controls and MCI-AD. These results did not change when calculating the dominant frequency from the occipital electrodes instead of using all electrodes. Dominant frequency variability was not significantly different between the groups.

The results did not change when restricting the analysis to the 21 electrodes that are part of the 10-20 system (see Supplementary Table S1).

The regional analysis showed very similar results compared to the analysis where all electrodes were combined (see Supplementary Table S2). The only slight difference was that alpha power was significantly reduced in MCI-LB compared to MCI-AD patients in posterior (*p* = 0.047) and lateral (*p* = 0.01) regions whereas mean alpha power from all electrodes was not significantly different between the two MCI groups (*p* = 0.07).

### ROC analysis for diagnostic discrimination

Area under the ROC curve (AUC) values, sensitivity, and specificity for the different EEG measures for differentiating between MCI-LB and MCI-AD are shown in Table 3. Beta power and dominant frequency achieved the highest AUC values of 0.71 and 0.70, respectively. Specificity was high for some measures (up to 0.97 for alpha power); however, sensitivity was generally much lower.

**Table 3:** Results from receiver operating characteristic (ROC) analysis to distinguish MCI-AD from MCI-LB

EEG measure	AUC [95% CI]	Cutoff for MCI-LB	Sensitivity	Specificity
Delta power	0.54 [0.41, 0.67]	> 21.9	0.23	0.89
Theta power	0.60 [0.47, 0.73]	> 10.7	0.33	0.89
Pre-alpha power	0.68 [0.56, 0.81]	> 28.1	0.56	0.83
Alpha power	0.66 [0.53, 0.78]	< 20.5	0.41	0.97
Beta power	0.71 [0.59, 0.83]	< 19.0	0.61	0.81
Theta/alpha ratio	0.64 [0.51, 0.77]	> 0.56	0.49	0.83
DF, all electrodes	0.70 [0.58, 0.82]	< 7.1	0.51	0.86
DF, occipital electrodes	0.69 [0.57, 0.81]	< 7.1	0.51	0.86

The sensitivity/specificity cutoff was determined using Youden’s index

*AUC* area under the receiver operating curve, *CI* confidence interval, *DF* dominant frequency, *MCI-AD* mild cognitive impairment with Alzheimer’s disease, *MCI-LB* probable mild cognitive impairment with Lewy bodies

### Association with clinical symptoms

**Fig. 4:**
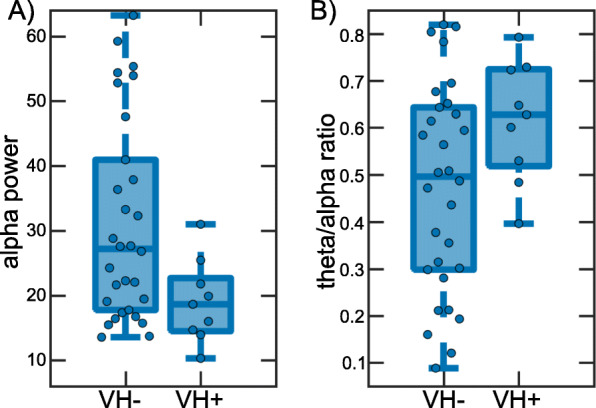
Comparison of quantitative EEG characteristics between patients with and without visual hallucinations. **a** Comparison of alpha power and **b** theta/alpha ratio between MCI-LB patients with visual hallucinations (VH+, *N* = 9) and without visual hallucinations (VH−, *N* = 30). In each boxplot, the central line corresponds to the sample median; the upper and lower border of the box represent the 25th and 75th percentile, respectively; and the length of the whiskers is 1.5 times the interquartile range

There was a significant reduction in alpha power in MCI-LB patients with visual hallucinations (*N* = 9, mean (SD) = 19.1 (6.4)) compared to those without visual hallucinations (*N* = 30, mean (SD) = 31.1 (15.4); *t*_37_ = 2.5, *p* = 0.02; Fig. 4a). Additionally, the theta/alpha ratio was increased in patients with visual hallucinations (mean (SD) = 0.61 (0.13)) compared to patients without visual hallucinations (mean (SD) = 0.47 (0.22), *t*_24_ = 2.4, *p* = 0.02, Fig. 4b).

None of the EEG measures showed a significant difference between those MCI-LB patients with cognitive fluctuations (*N* = 21) compared to those without cognitive fluctuations (*N* = 18) or between MCI-LB patients taking cholinesterase inhibitors (*N* = 18) compared to those patients not taking cholinesterase inhibitors (*N* = 20, all *p* > 0.1). Similarly, EEG measures were not different between MCI-AD patients taking cholinesterase inhibitors (*N* = 7) compared to those MCI-AD patients not taking these medications (*N* = 27).

MCI-LB patients with more than two core symptoms/abnormal biomarkers had significantly higher pre-alpha power, lower alpha power, and a higher theta/alpha ratio compared to MCI-LB patients with only two core symptoms or one core symptom and one abnormal biomarker (Supplementary Table S3). Higher delta power, theta power, pre-alpha power, and theta/alpha ratio correlated with higher Lewy body symptom/biomarker count whereas higher alpha power, beta power, and dominant frequency correlated with lower Lewy body symptom/biomarker count (Supplementary Table S3). There were no significant differences in overall cognition (ACE-R scores) between MCI-LB patients with more than two core symptoms/abnormal biomarkers compared to patients with two symptoms, and the Lewy body symptom/biomarker count was not significantly correlated with the ACE-R score (see Supplementary Table S3).

More severe cognitive impairment (as measured by the ACE-R) was correlated with higher pre-alpha power, lower alpha power, and decreased dominant frequency in the MCI-AD group (Supplementary Table S4). In the MCI-LB group, more severe cognitive impairment correlated with higher theta power, higher pre-alpha power, lower alpha power, higher theta/alpha ratio, and decreased dominant frequency (Supplementary Table S4).

## Discussion

In this study, we investigated the differences in quantitative EEG measures between highly phenotyped patients with MCI-AD and MCI-LB and in comparison with similarly aged healthy controls. We showed that there are significant differences between the two MCI groups at the group level with more severe EEG abnormalities in MCI-LB compared to MCI-AD and that these EEG changes correlated with the burden of Lewy body features, supporting their high specificity for Lewy body disease. However, their diagnostic accuracy for differentiating MCI-LB from MCI-AD is modest and not consistent with previous studies [17].

Overall, the findings suggest a slowing of the EEG in MCI-LB patients compared to healthy controls by a shift in power from beta and alpha frequency bands towards slower frequencies in the pre-alpha and theta range. This was also reflected by a shift of the dominant frequency towards slower frequencies. These findings are consistent with the results from previous EEG studies at the dementia stage which generally report an increase in slow-wave activity and slowing of the dominant EEG rhythm in DLB [10, 18–20]. A slowing of the dominant frequency has also been reported in MCI-LB patients before [21].

We did not observe any group differences in dominant frequency variability which has previously been found to be increased in DLB patients compared to controls [12, 17, 19]. However, other studies have failed to replicate these findings [10, 18, 22], and our results further indicate that an increase in dominant frequency variability may not be a reliable feature of Lewy body disease.

The only difference between MCI-AD patients and healthy controls was an increase in pre-alpha power, suggesting only a small degree of EEG slowing. This is again consistent with previous reports at the dementia stage which have generally found less severe EEG abnormalities in AD compared to DLB [10, 12, 19]. Additionally, we found significantly increased pre-alpha power, decreased beta power, and slower dominant frequency in the MCI-LB compared to the MCI-AD group, which indicates more severe EEG slowing in MCI-LB than in MCI-AD patients and mirrors the findings in dementia patients. Furthermore, we found more severe EEG abnormalities in MCI-LB patients with more core Lewy body symptoms and/or abnormal biomarkers and a higher symptom count correlated with more severe EEG slowing. This indicates that there is an association between more widespread Lewy body disease and more severe EEG abnormalities and suggests that Lewy body disease might affect electro-cortical activity in a dose-dependent manner.

While there were significant group-level differences in EEG characteristics between the two MCI groups, it is difficult to translate this, at present, into a measure for use in early clinical diagnosis. Power in the beta frequency range and dominant frequency had the highest diagnostic accuracy; however, they only reached moderate AUC values of around 0.7. The specificity for MCI-LB was very high for certain EEG measures (up to 0.97 for alpha power with a cutoff of < 20.5) which suggests that if a substantial shift of power towards slower frequencies is observed, i.e., in the case of a very abnormal EEG, a diagnosis of MCI-LB over MCI-AD becomes highly likely. The relationship between EEG slowing and Lewy body symptom count (see above) suggests that these might be the MCI-LB patients with more widespread Lewy body disease and these are easier to distinguish from MCI-AD patients based on their more severe EEG slowing. However, sensitivity was generally much lower, i.e., in the case of a more normal EEG, differentiating between MCI-AD and MCI-LB is difficult and many MCI-LB cases would be missed by applying these measures for diagnostic purposes. These findings thus show that early changes in quantitative EEG characteristics are specific, but not sensitive to Lewy body disease which is in alignment with the diagnostic performance of other biomarkers such as FP-CIT and MIBG in early Lewy body disease [8].

In a previous EEG study in MCI patients, Bonanni et al. [17] found that all MCI patients who converted to DLB within a 3-year follow-up period had an abnormal EEG whereas 93% of MCI patients who developed AD within the follow-up period had normal EEGs. The high specificity of EEG abnormalities for MCI-LB in this report is in line with our findings whereas the high sensitivity stands in contrast to the findings of the present study. However, the sample in this previous study was more selective by only including patients who developed dementia within 3 years and might therefore be biased towards MCI patients with more severe disease and thus having a rapid progression to dementia. When also taking into account those MCI patients who did not convert to dementia within 3 years, diagnostic accuracy was reduced to an overall predictive value of 76% [17]. Furthermore, in a multi-center study in dementia patients, the diagnostic accuracy of EEG was also found to be much lower [19], indicating that heterogeneity in EEG acquisition and analysis protocols across different centers might be an important limiting factor.

Another previous EEG study that attempted to differentiate MCI-AD from MCI-LB did not find any EEG measures that reliably distinguished between the two MCI subgroups [21]. However, this was a retrospective study of selected MCI-AD and MCI-LB patients who were recruited using clinical criteria which differed across the several centers who supplied patient data. Furthermore, participants from the different centers were also assessed using different EEG protocols which again makes the comparison difficult [21].

We explored the influence of the presence of visual hallucinations on quantitative EEG characteristics in MCI-LB patients and found that early EEG slowing, both in terms of a reduction of power in the alpha frequency range and a shift of power towards slower frequencies, might be more severe in patients who experience visual hallucinations compared to those patients who do not have visual hallucinations, a finding which has previously been reported in dementia patients [14]. However, the number of patients experiencing visual hallucinations in our cohort was small (*N* = 9) and these findings should therefore be interpreted with caution, and replication in a larger group of patients with a higher occurrence of visual hallucinations is needed.

### Limitations

Strengths of the present study include consistent and robust clinical assessments, diagnoses by a consensus panel, the use of two diagnostic biomarkers, and the prospective longitudinal design of the study. A potential limitation is the use of acetylcholinesterase inhibitors in the MCI patients as more patients in the MCI-LB group were taking these medications compared to the MCI-AD group. In the north east of England, services implement expert advice and thus have high prescription rates for DLB/MCI-LB patients because neuropsychiatric symptoms are treated using anti-dementia drugs early on. It has been shown that cholinergic medication can normalize EEG measures [15, 16], and it is therefore possible that differences between the two MCI groups were occluded by the higher number of MCI-LB patients taking these medications. However, when comparing MCI-LB patients who were taking acetylcholinesterase inhibitors to those who were not, we did not find any significant differences in any EEG measures. The same was found in the MCI-AD patients although this result should be interpreted with caution because of the small number of MCI-AD patients who were taking acetylcholinesterase inhibitors (*N* = 7). Furthermore, it would be unethical to withdraw medication, and including medicated patients is more reflective of clinical practice. Another potential limitation is the lack of AD biomarkers in our study.

## Conclusions

In conclusion, the present study suggests that early EEG slowing is specific to MCI-LB and, from a diagnostic point of view, a very abnormal EEG favors MCI-LB over MCI-AD. However, the overlap between the two MCI groups is large, and for patients with a more normal EEG, it is difficult to distinguish between the two MCI groups based on EEG characteristics alone. Given its specificity for Lewy body disease burden, EEG may be a promising future diagnostic biomarker and may have value as part of a panel of other biomarkers. To investigate this further, there needs to be a consensus regarding the standardization of acquisition protocols, analysis approaches, and choice of EEG parameters. Work in this field will undoubtedly yield benefits similar to those seen with CSF biomarkers for example [23]. More complex EEG analyses such as functional connectivity or source reconstruction methods may provide better sensitivity for detecting differences between the two MCI groups as well as between MCI-AD patients and controls [21, 24]. Furthermore, multimodal approaches including EEG features in addition to MIBG/FP-CIT or structural MRI markers may be another route by which it will be possible to achieve better accuracy for the early distinction between AD and DLB at the MCI stage.

## Supplementary information

**Additional file 1: Table S1.** Group comparison of quantitative EEG characteristics, restricting the analysis to 21 electrodes from the 10-20 system. **Table S2.** Group comparison of quantitative EEG characteristics, splitting the set of electrodes into four macroscopic regions. **Table S3.** Association between Lewy body symptom severity and EEG characteristics in the MCI-LB group. Two-sample t-tests comparing EEG measures between MCI-LB patients with two symptoms/biomarkers (*N*=13) and MCI-LB patients with more than two symptoms/biomarkers (*N*=26) and Spearman’s correlations between EEG characteristics and symptom/biomarker count (ranging from 2 to 6). *P*-values are FDR-corrected for multiple comparisons. **Table S4.** Association between the severity of overall cognitive impairment (ACE-R scores) and EEG characteristics in the MCI-LB and MCI-AD groups using Spearman’s correlations. P-values are FDR-corrected for multiple comparisons.

## Data Availability

The data that support the findings of this study are available from the corresponding author, upon reasonable request.

## Abbreviations

ACE-R: Addenbrooke’s Cognitive Examination–Revised
AD: Alzheimer’s disease
AUC: Area under the ROC curve
CAF: Clinician assessment of fluctuations
CDR: Clinical Dementia Rating
CIRS-G: Cumulative Illness Rating Scale for Geriatrics
DCFS: Dementia Cognitive Fluctuations Scale
DLB: Dementia with Lewy bodies
EEG: Electroencephalography
ESS: Epworth Sleepiness Scale
FDR: False discovery rate
FP-CIT: ^123^I-*N*-fluoropropyl-2β-carbomethoxy-3β-(4-iodophenyl) single-photon emission computed tomography
GDS: Geriatric Depression Scale
IADL: Instrumental Activities of Daily Living
MCI-AD: Mild cognitive impairment with Alzheimer’s disease
MCI-LB: Mild cognitive impairment with Lewy bodies
MIBG: ^123^Iodine-metaiodobenzylguanidine myocardial scintigraphy
MSQ: Mayo Sleep Questionnaire
MMSE: Mini-Mental State Examination
NEVHI: North-East Visual Hallucinations Interview
NPI: Neuropsychiatric Inventory
PSD: Power spectral density
ROC: Receiver operating characteristic
UPDRS-III: Unified Parkinson’s Disease Rating Scale motor sub-score
