# Supplementary material for: Quantitative EEG as a biomarker in mild cognitive impairment with Lewy bodies
Source: Alzheimers Res Ther. 2020 Jul 8;12:82. doi: 10.1186/s13195-020-00650-1 (PMC7346501; doi:10.1186/s13195-020-00650-1)
Supplement: Supplementary file 1 — Additional file 1: Table S1. Group comparison of quantitative EEG characteristics, restricting the analysis to 21 electrodes from the 10-20 system. Table S2. Group comparison of quantitative EEG characteristics, splitting the set of electrodes into four macroscopic regions. Table S3. Association between Lewy body symptom severity and EEG characteristics in the MCI-LB group. Two-sample t-tests comparing EEG measures between MCI-LB patients with two symptoms/biomarkers (N=13) and MCI-LB patients with more than two symptoms/biomarkers (N=26) and Spearman’s correlations between EEG characteristics and symptom/biomarker count (ranging from 2 to 6). P-values are FDR-corrected for multiple comparisons. Table S4. Association between the severity of overall cognitive impairment (ACE-R scores) and EEG characteristics in the MCI-LB and MCI-AD groups using Spearman’s correlations. P-values are FDR-corrected for multiple comparisons. [file 13195_2020_650_MOESM1_ESM.docx]

Supplementary Material:

Quantitative EEG as a biomarker in mild cognitive impairment with Lewy bodies

Julia Schumacher, John-Paul Taylor, Calum A. Hamilton, Michael Firbank, Ruth A. Cromarty, Paul C. Donaghy, Gemma Roberts, Louise Allan, Jim Lloyd, Rory Durcan, Nicola Barnett, John T. O’Brien, Alan J. Thomas

Correspondence: julia.schumacher@newcastle.ac.uk

Supplementary Table S1**:** Group comparison of quantitative EEG characteristics, restricting the analysis to 21 electrodes from the 10-20 system.

|  | HC | MCI-AD | MCI-LB | Group comparison |
| --- | --- | --- | --- | --- |
|  |  |  |  |  |
| Delta power | 14.6 | 14.3 | 15.2 | F(2,102)=0.5, p=0.59 |
|  | [11.7, 17.5] | [12.0, 16.6] | [12.9, 17.5] |  |
| Theta power | 5.9 | 7.1 | 8.8 | F(2,102)=4.4, p=0.01 |
|  | [4.7, 7.0] | [6.1, 8.2] | [7.3, 10.4] | p(HC,MCI-AD)=0.22  p(HC,MCI-LB)=0.006  p(MCI-AD,MCI-LB)=0.51 |
| Pre-alpha power | 13.1 | 19.5 | 28.6 | F(2,102)=15.8, p<0.001 |
|  | [9.8, 16.5] | [16.0, 23.0] | [24.0, 33.3] | p(HC,MCI-AD)=0.006  p(HC,MCI-LB)<0.001  p(MCI-AD,MCI-LB)=0.02 |
| Alpha power | 39.7 | 33.5 | 28.2 | F(2,102)=5.4, p=0.006 |
|  | [33.8, 45.5] | [29.1, 37.9] | [23.4, 32.9] | p(HC,MCI-AD)=0.42  p(HC,MCI-LB)=0.001  p(MCI-AD,MCI-LB)=0.07 |
| Beta power | 26.4 | 25.2 | 18.9 | F(2,102)=7.8, p=0.001 |
|  | [22.6, 30.2] | [22.3, 28.1] | [15.3, 22.5] | p(HC,MCI-AD)=1.0  p(HC,MCI-LB)=0.001  p(MCI-AD,MCI-LB)=0.001 |
| Theta/alpha ratio | 0.34 | 0.42 | 0.51 | F(2,102)=7.1, p<0.001 |
|  | [0.28, 0.41] | [0.36, 0.48] | [0.44, 0.58] | p(HC,MCI-AD)=0.24  p(HC,MCI-LB)=0.001  p(MCI-AD,MCI-LB)=0.12 |
| DF, all electrodes | 8.4 | 8.0 | 7.2 | F(2,102)=9.0, p<0.001 |
|  | [8.0, 8.9] | [7.6, 8.3] | [6.8, 7.6] | p(HC,MCI-AD)=0.19  p(HC,MCI-LB)<0.001  p(MCI-AD,MCI-LB)=0.02 |
| DF, occipital electrodes | 8.5 | 7.9 | 7.3 | F(2,102)=8.5, p<0.001 |
|  | [8.1, 9.0] | [7.5, 8.3] | [6.9, 7.7] | p(HC,MCI-AD)=0.09  p(HC,MCI-LB)<0.001  p(MCI-AD,MCI-LB)=0.052 |
| DFV, occipital electrodes | 1.4 | 1.5 | 1.1 | F(2,102)=3.4, p=0.02 |
|  | [1.0, 1.7] | [1.3, 1.8] | [0.9, 1.3] | p(HC,MCI-AD)=0.26  p(HC,MCI-LB)=1.0  p(MCI-AD,MCI-LB)=0.05 |
| Mean [95% confidence interval] of different quantitative EEG characteristics. Group comparisons were performed using univariate ANOVAs followed by post-hoc tests, Bonferroni-corrected for multiple comparisons. Sex was included as a covariate.  DF, dominant frequency; DFV, dominant frequency variability; HC, healthy controls; MCI-AD, mild cognitive impairment with Alzheimer’s disease; MCI-LB, probable mild cognitive impairment with Lewy bodies | | | | |

Supplementary Table S2**:** Group comparison of quantitative EEG characteristics, splitting the set of electrodes into four macroscopic regions.

|  | | HC | MCI-AD | MCI-LB | Group comparison |
| --- | --- | --- | --- | --- | --- |
| Delta power | |  |  |  | Main effect diagnosis: F(2,102)=0.8, p=0.47  Interaction diagnosis*region: F(5,242)=2.7, p=0.02 |
|  | frontal | 16.3 (8.2) | 15.4 (7.2) | 16.0 (7.2) | F(2,102)=0.6, p=0.6 |
|  | central | 13.7 (7.7) | 14.0 (6.7) | 14.6 (6.5) | F(2,102)=1.3, p=0.3 |
|  | lateral | 14.7 (7.2) | 14.5 (6.5) | 15.9 (7.1) | F(2,102)=0.4, p=0.7 |
|  | posterior | 12.1 (8.3) | 12.5 (6.7) | 13.5 (7.3) | F(2,102)=1.1, p=0.3 |
| Theta power | |  |  |  | Main effect diagnosis: F(2,102)=4.7, p=0.01  Interaction diagnosis*region: F(5,249)=4.7, p<0.001 |
|  | frontal | 6.4 (3.4) | 7.7 (3.4) | 9.2 (5.1) | F(2,102)=3.5, p=0.03 |
|  |  |  |  |  | p(HC,MCI-AD)=0.3, p(HC-MCI-LB)=0.02,  p(MCI-AD, MCI-LB)=0.8 |
|  | central | 5.6 (2.9) | 6.8 (3.0) | 8.5 (4.4) | F(2,102)=5.2, p=0.007 |
|  |  |  |  |  | p(HC,MCI-AD)=0.2, p(HC-MCI-LB)=0.003,  p(MCI-AD, MCI-LB)=0.4 |
|  | lateral | 5.9 (2.8) | 6.7 (2.7) | 8.6 (4.5) | F(2,102)=5.6, p=0.001 |
|  |  |  |  |  | p(HC,MCI-AD)=0.09, p(HC-MCI-LB)=0.003,  p(MCI-AD, MCI-LB)=0.7 |
|  | posterior | 5.2 (3.5) | 6.8 (3.3) | 8.8 (5.5) | F(2,102)=3.8, p=0.025 |
|  |  |  |  |  | p(HC,MCI-AD)=0.6, p(HC-MCI-LB)=0.007,  p(MCI-AD, MCI-LB)=0.2 |
| Pre-alpha | |  |  |  | Main effect diagnosis: F(2,102)=16.2, p<0.001  Interaction diagnosis*region: F(5,258)=0.9, p=0.5 |
|  | frontal | 13.3 (10.0) | 19.4 (10.2) | 28.0 (14.4) |  |
|  | central | 12.7 (8.6) | 18.6 (9.6) | 27.3 (13.4) |  |
|  | lateral | 13.2 (7.8) | 20.3 (10.7) | 29.2 (14.2) |  |
|  | posterior | 13.7 (10.3) | 20.4 (11.4) | 30.8 (15.9) |  |
| Alpha | |  |  |  | Main effect diagnosis: F(2,102)=5.8, p=0.004  Interaction diagnosis*region: F(6,285)=3.9, p=0.001 |
|  | frontal | 37.0 (16.6) | 31.4 (13.7) | 26.6 (14.0) | F(2,102)=4.4, p=0.02 |
|  |  |  |  |  | p(HC,MCI-AD)=0.5, p(HC-MCI-LB)=0.003,  p(MCI-AD, MCI-LB)=0.1 |
|  | central | 38.3 (14.8) | 32.7 (11.8) | 28.4 (13.4) | F(2,102)=4.5, p=0.01 |
|  |  |  |  |  | p(HC,MCI-AD)=0.4, p(HC-MCI-LB)=0.003,  p(MCI-AD, MCI-LB)=0.2 |
|  | lateral | 39.0 (12.8) | 33.7 (10.9) | 27.3 (13.7) | F(2,102)=7.8, p=0.001 |
|  |  |  |  |  | p(HC,MCI-AD)=0.4, p(HC-MCI-LB)<0.001,  p(MCI-AD, MCI-LB)=0.01 |
|  | posterior | 44.6 (18.4) | 36.6 (14.7) | 30.4 (17.9) | F(2,102)=6.2, p=0.003 |
|  |  |  |  |  | p(HC,MCI-AD)=0.3, p(HC-MCI-LB)<0.001,  p(MCI-AD, MCI-LB)=0.047 |
| Beta | |  |  |  | Main effect diagnosis: F(2,102)=8.3, p<0.001  Interaction diagnosis*region: F(5,263)=3.6, p=0.003 |
|  | frontal | 26.5 (10.6) | 25.8 (8.9) | 19.9 (11.9) | F(2,102)=6.4, p=0.002 |
|  |  |  |  |  | p(HC,MCI-AD)=1.0, p(HC-MCI-LB)=0.003,  p(MCI-AD, MCI-LB)=0.003 |
|  | central | 29.3 (10.9) | 27.6 (9.2) | 20.8 (11.2) | F(2,102)=7.9, p<0.001 |
|  |  |  |  |  | p(HC,MCI-AD)=1.0, p(HC-MCI-LB)<0.001,  p(MCI-AD, MCI-LB)=0.001 |
|  | lateral | 26.9 (9.0) | 24.4 (8.3) | 18.6 (10.1) | F(2,102)=7.7, p<0.001 |
|  |  |  |  |  | p(HC,MCI-AD)=0.9, p(HC-MCI-LB)<0.001,  p(MCI-AD, MCI-LB)=0.002 |
|  | posterior | 24.1 (11.5) | 23.4 (9.4) | 16.2 (11.3) | F(2,102)=7.3, p<0.001 |
|  |  |  |  |  | p(HC,MCI-AD)=1.0, p(HC-MCI-LB)<0.001,  p(MCI-AD, MCI-LB)<0.001 |
| Mean (standard deviation) of different quantitative EEG characteristics. Group comparisons were performed using repeated measures ANOVAs followed by univariate ANOVAs if the interaction between diagnosis and region was significant. Sex was included as a covariate.  HC, healthy controls; MCI-AD, mild cognitive impairment with Alzheimer’s disease; MCI-LB, probable mild cognitive impairment with Lewy bodies | | | | | |

Supplementary Table S3: Association between Lewy body symptom severity and EEG characteristics in the MCI-LB group. Two-sample t-tests comparing EEG measures between MCI-LB patients with two symptoms/biomarkers (N=13) and MCI-LB patients with more than two symptoms/biomarkers (N=26) and Spearman’s correlations between EEG characteristics and symptom/biomarker count (ranging from 2 to 6). P-values are FDR-corrected for multiple comparisons.

| EEG measure, mean (SD) | MCI-LB with 2 symptoms/ biomarkers (N=13) | MCI-LB with  > 2 symptoms/ biomarkers (N=26) | T-test | Correlation with symptom count |
| --- | --- | --- | --- | --- |
| Delta power | 12.0 (5.5) | 16.4 (7.0) | t_37_=2.1, p_FDR_=0.06 | ρ=0.33, p_FDR_=0.04 |
| Theta power | 7.2 (5.2) | 9.6 (4.5) | t_37_=2.1, p_FDR_=0.16 | ρ=0.40, p_FDR_=0.01 |
| Pre-alpha power | 18.3 (9.2) | 34.0 (13.4) | t_37_=3.7, p_FDR_=0.004 | ρ=0.54, p_FDR_=0.004 |
| Alpha power | 40.0 (17.7) | 22.5 (8.6) | t_37_=3.7, p_FDR_=0.004 | ρ=-0.43, p_FDR_=0.01 |
| Beta power | 22.2 (9.3) | 17.2 (11.6) | t_37_=1.8, p_FDR_=0.08 | ρ=-0.44, p_FDR_=0.01 |
| Theta/alpha ratio | 0.38 (0.24) | 0.57 (0.17) | t_37_=2.9, p_FDR_=0.02 | ρ=0.44, p_FDR_=0.01 |
| DF, all electrodes | 7.8 (1.2) | 6.9 (1.0) | t_37_=2.3, p_FDR_=0.06 | ρ=-0.45, p_FDR_=0.01 |
| DF, occipital electr. | 7.8 (1.2) | 7.0 (1.1) | t_37_=2.2, p_FDR_=0.06 | ρ=-0.48, p_FDR_=0.01 |
| ACE-R | 86.8 (7.1) | 82.3 (9.9) | t_37_=1.6, p_FDR_=0.15 | ρ=-0.17, p_FDR_=0.30 |
| DF, dominant frequency; FDR, false discovery rate; MCI-LB, probable mild cognitive impairment with Lewy bodies; SD, standard deviation | | | | |

Supplementary Table S4: Association between the severity of overall cognitive impairment (ACE-R scores) and EEG characteristics in the MCI-LB and MCI-AD groups using Spearman’s correlations. P-values are FDR-corrected for multiple comparisons.

|  | Correlation with ACE-R scores in MCI-AD | Correlation with ACE-R scores in MCI-LB |
| --- | --- | --- |
| Delta power | ρ=-0.12, p_FDR_=0.51 | ρ=-0.23, p_FDR_=0.21 |
| Theta power | ρ=-0.21, p_FDR_=0.26 | ρ=-0.35, p_FDR_=0.046 |
| Pre-alpha power | ρ=-0.44, p_FDR_=0.02 | ρ=-0.37, p_FDR_=0.04 |
| Alpha power | ρ=0.40, p_FDR_=0.03 | ρ=0.50, p_FDR_=0.008 |
| Beta power | ρ=0.11, p_FDR_=0.51 | ρ=0.30, p_FDR_=0.09 |
| Theta/alpha ratio | ρ=-0.32, p_FDR_=0.08 | ρ=-0.48, p_FDR_=0.008 |
| DF, all electrodes | ρ=0.50, p_FDR_=0.008 | ρ=0.43, p_FDR_=0.02 |
| DF, occipital electr. | ρ=0.49, p_FDR_=0.008 | ρ=0.48, p_FDR_=0.008 |
| ACE-R, Addenbrooke’s Cognitive Examination – Revised; DF, dominant frequency; FDR, false discovery rate corrected; MCI-AD, mild cognitive impairment with Alzheimer’s disease; MCI-LB, mild cognitive impairment with Lewy bodies. | | |
